# Epidemiological, Clinical, and Histopathological Features of Breast Cancer in Haiti

**DOI:** 10.1200/JGO.17.00135

**Published:** 2018-02-13

**Authors:** Vincent DeGennaro, Faiz Jiwani, Elizabeth Patberg, Martin Gibbs, Rachel Libby, Dieudina Gabriel, Coy D. Heldermon, Karen Daily, Joseph Bernard

**Affiliations:** **Vincent DeGennaro Jr**, **F. Faiz Jiwani**, **Martin Gibbs**, **Dieudina Gabriel**, and **Joseph Bernard Jr**, Innovating Health International, Port-au-Prince, Haiti; **Vincent DeGennaro Jr**, **Martin Gibbs**, **Coy D. Heldermon**, and **Karen Daily**, University of Florida College of Medicine, Gainesville, FL; **F. Faiz Jiwani**, Baylor College of Medicine, Houston, TX; and **Elizabeth Patberg** and **Rachel Libby**, Emory School of Medicine, Atlanta, GA.

## Abstract

**Purpose:**

Little is known about the epidemiology of breast cancer in developing countries, and Haiti has perhaps the least data of any country in the Western Hemisphere.

**Methods:**

We conducted a retrospective review of all patients enrolled in an ongoing breast cancer treatment program in Port-au-Prince, Haiti, from July 1, 2013, through June 30, 2017. Data were drawn from each patient's electronic medical record, paper chart, and biopsy results.

**Results:**

The records of 525 women with breast cancer were reviewed for this study. The median age at presentation was 49 years (n = 507). The risk factors observed were as follows: postmenopausal, 50.8% (n = 354); nulliparity, 15.7% (n = 338); hormonal contraception use, 35.0% (n = 309); never breastfed, 20.6% (n = 316); family history of any cancer, 22.0% (n = 295); overweight, 51.5% (n = 332); and smoking, 5.0% (n = 338). Of all those staged, 83.9% (n = 447) of the patients presented with stage III/IV disease and more than half delayed care for > 12 months after first noticing a breast mass. For the subset of tumors for which estrogen receptor (ER; n = 245) and human epidermal growth factor receptor 2 (HER2; n = 179) status was available, the prevalence of ER-positive tumors was 51.8%, of HER2-positive tumors was 19.6%, and of triple-negative tumors was 38.5%. The 12-month mortality rate (n = 425) was 18.4% overall and 27.5% for those who presented with stage IV disease. Median survival was not reached.

**Conclusion:**

Breast cancer in Haiti presents at an early age and advanced stage. Triple-negative, ER-negative, and high-grade tumors are common. Delays in seeking care and incomplete treatment likely contribute to the high mortality rate; however, as in black women in the United States, the distribution of tumor types may contribute to disparate outcomes.

## INTRODUCTION

Annually, more than 1.5 million women are diagnosed with breast cancer globally and 521,000 die of the disease.^1,2^ In 2010, 51% of newly diagnosed breast cancers occurred in low- and middle-income countries (LMICs) and a near doubling of incident cases is expected by 2030.^3^ Although the highest prevalence of disease is in developed nations, the incidence of breast cancer is increasing at a faster rate in LMICs.^4^

Women in LMICs experience disease at an earlier age and have a higher mortality rate,^5^ likely due to a combination of socioeconomic factors and a more aggressive tumor pathology. The median age of death resulting from breast cancer in the developing world is 54 years, whereas in the United States, it is 62 years.^6^ The age-standardized mortality rates from breast cancer in developing nations are three times that of developed countries.^7^ Adesunkanmi et al^8^ found an average of 11.2 months’ delay between onset of symptoms and presentation in Nigeria. Thirty-nine percent of the 212 Nigerian women in their study presented with fungating breast masses. In LMICs, it is estimated that 75% of newly diagnosed breast cancers are stage III or IV, but data are sparse, particularly in low-income countries.^9,10^ In certain populations, the incidence of triple-negative tumors is much higher than in the developed world. For example, in one study of 507 women from Nigeria and Senegal, most of the women had triple-negative breast pathology^11^; in a study of 231 Nigerian women, almost half had triple-negative disease.^12^

In a systematic review of breast cancer epidemiology worldwide from 1980 through 2010, 41 countries had no site-specific data available and few had national cancer registries.^13^ On the basis of epidemiologic models from similar countries, the incidence of breast cancer in Haiti is estimated to be 23.9 per 100,000 women and the prevalence, according to GLOBOCAN 2012, is 88.4 per 100,000 women,^14^ with an approximate relative mortality rate of 45%.^15^ In contrast, the United States has a higher incidence of breast cancer (121 per 100,000 women) but a much lower relative mortality rate (19%).^16^ Based on these data and our anecdotal experience in Haiti, we hypothesized that breast cancer affects women at younger ages, is more frequently triple-negative breast cancer, and patients in Haiti present with later-stage disease.

## METHODS

We conducted a retrospective review of all patients enrolled over a 4-year period in an ongoing breast cancer treatment program in Port-au-Prince, Haiti. The clinical and research programs are supported through a collaboration of the US-based nonprofit Innovating Health International (IHI) and the University of Florida College of Medicine Department of Medicine. The IHI Women’s Cancer Center was located at Hospital Bernard Mevs in Port-au-Prince from July 1, 2013, until April 30, 2016, and then at St Luke’s Hospital in Port-au-Prince. The IHI center is one of four chemotherapy programs in Haiti and is the second largest in terms of patient volume.

From July 2013 to June 2017, 525 women presented to the outpatient Women’s Cancer Center for evaluation with biopsy specimen–confirmed breast carcinoma and were included in this review. A password-protected database held patients’ personal information and study data. All data gathering was completed primarily by Haitian and American medical students according to a standardized key. In total, partial or complete information was gathered from 525 patients; relevant files for an additional 27 patients were unable to be found. We collected demographic and clinicopathological data from the paper and electronic medical records at the center as well as those from the hospitals, which were separate from the clinic charts. Clarification of incomplete, undecipherable, or missing information was mediated via consensus of the oncology team at the Women’s Cancer Center.

The inclusion criterion was a biopsy specimen demonstrating a breast carcinoma among patients who presented to the IHI Women’s Cancer Center from July 1, 2013, to June 30, 2017. There is a total of 525 women met that criterion; however, the rest of the categories measured report on varying numbers of patients for several reasons, including cost issues, incomplete clinical information, partial documentation by providers, or lack of treatment options in Port-au-Prince before July 2013. For example, because immunohistochemistry is not available in Haiti, 245 of the 525 women with positive biopsy specimens had a reportable estrogen receptor (ER) status and 179 had known human epidermal growth factor receptor 2 (HER2) status.

Tumor staging was conducted according to the American Joint Committee on Cancer system^17^ at the earliest recorded presentation to a physician. If a patient received treatment before presenting to our clinic, the tumor stage was assigned using information from original presentation whenever clinically documented and available at the time of review. Most patients were staged clinically with physical examination, chest radiograph, and liver ultrasound; some underwent computed tomography scans when clinically and financially available, in accordance with Haiti’s national staging guidelines. Histologic tumor grading was performed using the Elston-Ellis Modified Histologic System.^18^ Immunohistochemistry was conducted using ER/PR pharmDx and HercepTest kits (both from Agilent Technologies, Santa Clara, CA).^19^ For HER2, 3+ is a positive result; 2+ is equivocal, which was confirmed by fluorescent in situ hybridization; and ≤ 1+ is a negative result.^20^

Study data were collected and managed using REDCap (Research Electronic Data Capture), a secure, Web-based electronic data capture application hosted at University of Florida. Patients’ ages were calculated from dates of birth in the chart, when available, and death surveillance occurred through regular contact with nursing staff. Where patients were confirmed to be deceased but the date of death was uncertain, the day of the most recent surveillance update was used as the date of death, which likely resulted in underestimation of survival. This study was approved by the Haitian Ministry of Health’s National Bioethics Committee located in Port-au-Prince, Haiti, as well as the institutional review board at the University of Florida College of Medicine.

## RESULTS

The personal characteristics of the patients with breast cancer are listed in Table 1. The median age on presentation to the center was 49.1 years. Most patients (83.9%) presented with advanced disease and 85% of those had a stage documented. The overall stage at presentation was ductal carcinoma in situ in 0.4%, stage I in 0.4%, stage II in 15.2%, stage III in 55.5%, and stage IV in 28.4%. More than half of the women with a documented clinical breast examination had skin and/or chest wall involvement on presentation. Of the 157 patients who died since enrollment in the treatment program, 42.7% (n = 67) had stage IV disease on presentation to the IHI chemotherapy clinic.

**Table 1:**
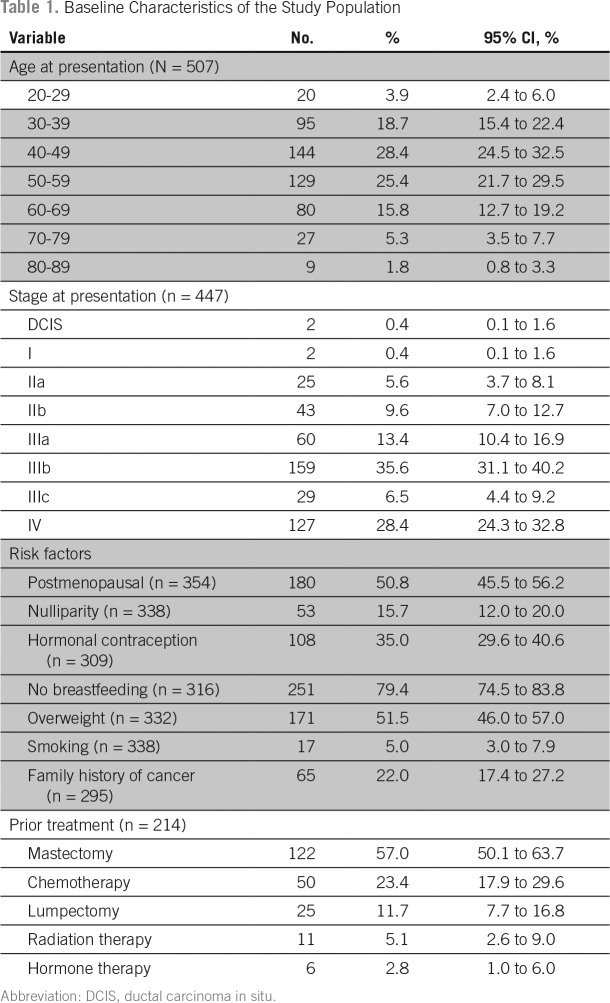
Baseline Characteristics of the Study Population

The prevalence of each of the risk factors we noted was as follows: postmenopausal state, 50.8% (n = 354); nulliparity, 15.7% (n = 338); hormonal contraception use, 35.0% (n = 309); never breastfed, 20.6% (n = 316); family history of any cancer, 22.0% (n = 295); overweight (body mass index > 25 kg/m^2^), 51.5% (n = 332); and smoking, 5.0% (n = 338). Most women (78.0%) had no self-reported family history of cancer, but of the 65 women who did report cancer in a family member, 27.7% (n = 18) reported breast cancer in a first-degree relative. At the time of presentation to our clinic, 88.7% (n =236) had a high functional capacity, as measured by an Eastern Cooperative Oncology Group performance score of 0 to 1. Based on those with a recorded body mass index (n = 332), 51.5% (n = 171) were overweight and 18.4% (n = 61) were obese.

Of the 430 women with data, 42.6% (n = 183) had received some form of treatment for their breast cancer at an outside facility before presentation at IHI. Most of them had a mastectomy (57.0%) and 23.4% had some chemotherapy. Most of these patients presented to IHI with a recurrence of disease after receiving some (ie, mastectomy only) or all of their treatment (ie, mastectomy and chemotherapy) at an outside facility in the remote past, although many had undergone mastectomy at an outside facility and presented to IHI for adjuvant chemotherapy and hormone therapy.

The pathologic features of breast cancer for the women included in this study are presented in Table 2. Invasive ductal carcinoma was seen in 87.3% of the 403 women for whom we had biopsy-specimen data. The percentage of tumors that were of histologic grade 1, 2, or 3 (n = 218) were 13.3%, 48.2%, 34.9%, respectively. For the subset of tumors for which ER (n = 245) and HER-2 (n = 179) status was available, the prevalence of ER-positive tumors was 51.8%, of HER-2/neu-positive tumors was 19.6%, and of triple-negative tumors was 38.5%. Most women in the study were from the Port-au-Prince metropolitan area, but 22.8% were referrals from distant parts of Haiti > 2 hours away. Of the subset of patients in the study who were being evaluated for breast cancer complaints for the first time (n = 247), half presented to IHI > 12 months after first noticing a breast mass. Only 34.2% (n = 125) of patients presented to a physician within 6 months of noticing a breast mass.

**Table 2:**
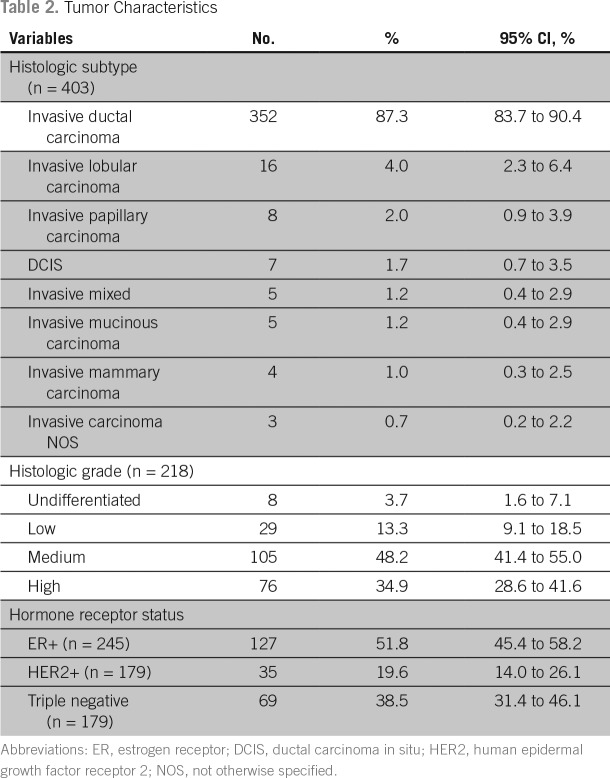
Tumor Characteristics

Median survival was not reached for the overall cohort. Median survival for those who died (n = 157) was 24 months when calculated by using the first date that the patient noticed the tumor in her breast until her death was noted by clinic staff either through clinical contact or during semiannual reviews. When calculated from the time of presentation until their death was noted by staff, the median survival was 14 months for those who died. The 12-month survival rate (n = 425) was 81.6% overall from the date of first consultation and 89.9% from when they first noted the tumor. For those who presented with stage IV disease, the 12-month survival rate from first consultation was 73.5% and the 24-month survival from first consultation was 28.2%. For those who presented with stage IV disease, the 12-month survival rate from first noting the tumor was 81.5% and the 24-month survival from first noting the tumor was 53.6%.

The median age at presentation became younger and the stage at presentation became earlier since implementation of an awareness program in late 2015. Neither was statistically significant, and it is too early for valuable mortality data for 2016 through 2017.

## DISCUSSION

Given that the center is the second largest cancer program by volume, is available regardless of patients’ economic means, and that nearly one-quarter of the patients come from > 2 hours away, the sample in this study is representative of the breast cancer epidemic in Haiti at large. The cohort of women presenting to IHI with breast cancer appeared to be younger and with more advanced disease than that seen in the United States, even when compared with black women. Although Haitian population data are unavailable to calculate age-adjusted rates for significant analysis, the trend in the Haiti cohort is, nonetheless, impressive. The cohort in Haiti demonstrates many of the known risk factors for worse prognosis: younger than age 50 years, black race, low socioeconomic stratum, and stage IV disease at presentation.^21-23^ Scarce risk-factor data exist in Haiti for comparison of our cohort with the general female population, except for a recent study that demonstrated a similar smoking prevalence of 5.0% and an overweight prevalence of 34.3%, which was lower than that of our cohort (51.5%).^24^

Haitians are 95% of primarily African ancestry: Much of the population is descended from slaves coming from West and Central Africa, such as Nigeria and Senegal, with some mixing of genes between indigenous populations and Northern Europeans, similar to African Americans.^25,26^ We offer comparisons with data from East African countries such as Malawi and Rwanda, on the basis of availability of data and similar racial composition and socioeconomic levels.

The median age of breast cancer onset in the Haitian cohort was 49.1 years, which is considerably younger than that of women as a whole in the United States (62 years) and that of black women in the United States (58 years),^27^ but similar to that seen in Malawi^28^ and Rwanda.^29^ A much greater percentage of cases occur in women older than 50 years in the United States (79%) than in Haiti (48.5%).^30^ Given that the life expectancy in Haiti is 62 years,^31^ competing mortality may play a role in this finding. Without true incidence data, the contribution of breast cancer mortality to overall mortality in Haiti is still unknown.

Most women in the Haitian cohort presented with stage III/IV disease (83.9%), which contributed to the high mortality rate observed. This is similar to rates of late-stage presentation in Nigeria and Rwanda, which were 51.4% and 76%, respectively,^10,12^ but lower than that in the United States.^32,33^ Breast cancer histology and grade in our Haitian cohort resembles that seen in black women in the United States and elsewhere, including the prevalence of high-grade tumors in the United States^32,33^ and Malawi.^27^

In the Haitian cohort, 51.8% of tumors were ER positive, which is significantly lower than the rate of 68% to 77% seen in the all-race US cohorts, although it approximates the prevalence of 52% in black women in the United States^32,33^ and is much higher than that found in Nigeria (27%).^12^ The lower rates of ER-positive tumors result in increased cost of treatment, because tamoxifen is cheaper than intravenous chemotherapy and also are associated with an increased mortality rate, as well.^34-36^ The percentage of triple-negative breast cancers (38.5%) in the Haitian cohort was significantly higher than that seen in the United States for all races (10% to 20%)^37-39^ but similar to that seen in black women in the United States (28% to 47%)^40,41^ and in Nigeria (49%).^12^ Because women with triple-negative breast cancer have a poorer prognosis when compared with patients with other breast cancer subtypes, the high rate of triple-negative disease probably has an important effect on breast cancer–related mortality in Haiti.^42,43^

A lower-than-expected prevalence of ER-positive tumors and a high, triple-negative prevalence could be a result of specimen handling in Haiti. Most specimens stay in formalin for longer than the recommended 72-hour maximum, although this typically results in overfixation and, therefore, an overestimation of ER prevalence. One study demonstrated a 99% concordance when specimens were overfixed.^44^ The quality of the formalin may also vary in Haiti and can affect ER staining. False-negative results can occur from problems with tissue cautery, decalcification, prolonged ischemic time, and inappropriate fixation, all of which are possible in Haiti’s low-resource setting.^19^

The data from our cohort indicate a high rate of HER2-positive tumors, which may account for some of the excess mortality. The rate of HER2-positive tumors (19.6%) in Haiti is higher than the overall prevalence of 15.3% seen in the United States and 12.2% in black women in the United States,^40^ but lower than the rate in Nigeria (30%).^12^ The high rate of HER2-positive tumors in Haiti could have important implications for planning national treatment strategies. Data from developing countries, including data from this study, indicate a significant need for affordable trastuzumab in countries with large populations of African descent. Trastuzumab, even when manufactured as a generic medication by companies in India and South Africa, costs > $1,200 per dose, placing it out of reach of most people in developing countries, even when using short-duration 9-week courses.^45^ Generic licensing for lower-income countries and pooled purchasing mechanisms that guarantee minimum purchases can lower the price further.

Our data indicate there are significant barriers to care for women with breast cancer in Haiti. Of the 247 women who had no treatment before presentation at IHI, half palpated a lump in the breast > 12 months before arrival at our clinic, which is similar to the time to presentation seen in Nigeria and Malawi.^8,27^ Our research has indicated that less than one-third of women know the symptoms or complications of breast cancer. Other barriers included lack of knowledge of testing and treatment centers, and the perceived cost of treatment (G. Tillyard, personal communication, January 2017). According to discussions with patients who were lost to follow-up but who could be contacted, the most frequent reasons for lack of follow-up were a decision to stop treatment because of adverse effects in those who had end-stage disease and a decision to seek care closer to home, even though that care did not include any antineoplastic therapy.

In Haiti, the combination of significant delays in seeking care, the innate biologic aggressiveness of the breast cancer subtypes, and major barriers to adequate treatment lead to late-disease presentations and early, unnecessary deaths.

Haitian women are presenting at a young age with late-stage disease. To improve this and increase access to treatment, capacity must be expanded to more hospitals in geographically distributed areas throughout countries. Training of doctors and nurses, and expanding pathology capabilities are essential to increasing access to cancer care. IHI is working with the Haiti’s Ministry of Health and others to expand treatment services around the country. To increase awareness and promote early presentation, IHI has partnered with the Haiti’s Ministry of Health and local nonprofit organizations to launch an awareness campaign for self-breast examinations, via grassroots community meetings, the Internet, and radio, including launching a Creole-language Web site (kanseayiti.com).

In addition to providing important details about breast cancer in Haiti, this study may also provide insight into breast cancer in other countries with populations of African descent. The weakness of this study is the sample size and the incomplete data set. The data set is incomplete because of the lack of national health care databases, limited resources, staff shortages, minimal Internet connectivity, and other issues that face health care providers in resource-poor settings.In an increasingly globalized world, where developing countries bear a huge portion of the cancer burden, it is important that we work to extend prevention, treatment, and palliative care options to all patients with cancer. Additional studies are needed in nearly all LMICs to identify and explain regional variations in the presentations and natural history of breast cancer and other common malignancies. It is our hope that through research, training, infrastructure building, and awareness, we can start to influence breast cancer mortality in Haiti and the rest of the developing world.
